# Antibacterial Properties of Polysulfone Membranes Blended with Arabic Gum

**DOI:** 10.3390/membranes9020029

**Published:** 2019-02-14

**Authors:** Souhir Sabri, Ahmad Najjar, Yehia Manawi, Nahla Omer Eltai, Asma Al-Thani, Muataz Ali Atieh, Viktor Kochkodan

**Affiliations:** 1 Qatar Environment and Energy Research Institute (QEERI) Hamad Bin Khalifa University (HBKU), P.O. Box 34110 Doha, Qatar; ymanawi@qf.org.qa (Y.M.); mhussien@hbku.edu.qa (M.A.A.); 2 College of Life and Health Sciences, Hamad Bin Khalifa University (HBKU), P.O. Box 34110 Doha, Qatar; 3 Biomedical Research Center (BRC), Qatar University (QU), Doha 2713, Qatar; nahla.eltai@qu.edu.qa (N.O.E.); aaja@qu.edu.qa (A.A.-T.)

**Keywords:** polysulfone membranes, arabic gum, biofouling, membrane hydrophilicity, surface charge

## Abstract

Polysulfone (PS) membranes blended with different loadings of arabic gum (AG) were synthesized using phase inversion method and the antibacterial properties of the synthesized membranes were tested using a number Gram-negative (*Escherichia coli*, *Klebsiella pneumonia* and *Pseudomonas aeruginosa*) and Gram-positive (*Staphylococcus* *aureus*) bacterial species. It was shown that AG addition to the dope polymer solutions essentially changed porous structure, hydrophilicity and zeta potential of the cast PS/AG membranes. These changes were due to the amphiphilic properties of AG macromolecules that contained negatively charged hydrophilic residues. A pronounced decrease in bacterial attachment was seen in the field emission scanning electron microscopy (FESEM) images for PS/AG membrane samples compared to both commercial (Microdyn-Nadir) and bare PS (without AG) membranes. AG loading dependent trend was observed where the prevention of bacterial colonization on the membrane surface was strongest at the highest (7 wt. %) AG loading in the casting solution. Possible mechanisms for the prevention of bacterial colonization were discussed. Significantly, the inhibition of bacterial attachment and growth on PS/AG membranes was observed for both Gram-positive and Gram-negative bacterial models, rendering these novel membranes with strong biofouling resistance attractive for water treatment applications.

## 1. Introduction

Fouling is a natural and costly phenomenon that affects a wide range of industrial sectors such as cooling water towers, heat exchange systems, drinking water systems (wells, filtration membranes, distribution systems, storage tanks, plumbing systems in buildings), wastewater treatment and seawater desalination [1,2,3,4]. It is defined as the deposition of unwanted materials on the surfaces [5]. In membrane based technologies related to water treatment, four different types of fouling have been described: (1) mineral precipitation on the membrane surfaces (typically with low solubility salts such as magnesium, calcium and barium salts), (2) colloidal or particulate matter fouling (i.e., clay, silt and humic particles) (3) organic fouling (i.e., proteins, polysaccharides, lipids, oil and humic acids) and (4) biofouling by microorganisms as extracellular polymeric substances (EPS) produced by bacteria [6]. Biofouling has been reported as a major problem specifically affecting pressure driven membrane processes [7,8,9]. It occurs when the microorganisms adhere, grow and secrete EPS on the surfaces leading to the formation of a thin layer of microbial biofilm [10]. The presence of organic soluble foulants including natural organic matter are usually a prerequisite for bacterial colonization, where the soluble particles serve as a food source for the microorganisms [11]. The formation of EPS attracts more micro-organisms through bacterial signals called quorum sensing (QS) [12].

Membrane biofouling causes a significant decrease in the water flux due to the decrease in membrane permeability. Consequently, higher operating pressures are required, which means a higher energy consumption is incurred. Furthermore, frequent membrane cleaning and replacement would be needed which adds to the maintenance and operating costs [13]. 

Biofouling in membranes takes place through a series of complex physicochemical and biological processes that are highly dependent on the membrane surface characteristics (hydrophobicity, electrokinetic charge and pore size), feed water composition (temperature, pH and presence of nutrients), microbial characteristics (size, cell surface hydrophobicity and charge) and operating conditions [9,14,15]. Biofouling control remains a big challenge in any membrane technology setting, since unlike other types of fouling, biofouling has proven difficult to overcome. Whilst other fouling problems can be combated using different chemical and physical pre-treatments, such strategies have inherent limitations of their own [13]. Nonetheless, existing strategies have been adopted to reduce membrane biofouling and enhance membrane permeability, such as feed water pre-treatment with biocides including chlorine, ozone and UV irradiation. However, chlorination and ozonation have been reported to produce undesirable carcinogenic compounds capable of permeation through the membrane [16,17]. Furthermore, ozonation of wastewaters leads to production of assimilable organic carbon providing a vital carbon source for bacterial growth [18]. 

Biological controls can also be used in order to mitigate biofouling processes, especially through the inhibition of QS signal which is involved in cellular communication for Gram-positive and Gram-negative bacteria [19,20]. QS signaling is a mechanism that controls the expression of specific genes responsible for bacterial behavior including biofilm, formation, swarming, motility and production of EPS [21]. Kim et al. [22] demonstrated that fouled RO membrane surfaces harbored 60% of bacterial species contributing to biofilm formation through N-acylated homoserine lactones (NHL) (QS signal), indicating that NHL inhibition could be a good approach to reduce biofouling [22]. The use of bacteriophages can also be employed for the inhibition of bacterial growth by lysing bacterial cells and using them as hosts for replication [23]. It was demonstrated that some bacteriophages were successfully used in membrane bioreactors to reduce membrane biofouling and increase membrane permeability. However, QS signal inhibition and bacteriophage employment remain a big challenge due to their application at industrial scale and require further investigations [24,25]. 

Membrane cleaning is commonly adopted to reduce membrane biofouling and enhancing the membrane permeability. This approach consists of applying physical methods (including flushing, air sparging and ultrasound) or chemical methods (alkalis, acids, metal chelating agents, surfactants and enzymes) in order to kill microorganisms [26]. However, microorganisms can easily regrow via their utilization of the dead biomass as a support, which provides favorable conditions for bacterial growth [27]. Finally, membrane surface modification (polymer blending, grafting, coating or using inorganic compounds or antimicrobial additives) has be shown to greatly alter the surface properties and is capable of confer antimicrobial properties to the membrane surface [28,29]. 

It was recently demonstrated that arabic gum (AG) extract effectively improved the properties of a polyethersulfone (PES) membrane such as surface charge, hydrophilicity, porosity and permeate flux, as well as resistance to biofouling [30]. However the antifouling properties of polysulfone (PS)/AG membranes were tested solely with *Escherichia coli (E*. *coli*) bacteria [31]. 

In the current study, novel PS membranes with different AG loadings were fabricated and characterized using scanning electron microscopy (SEM), contact angle and zeta potential measurements. The antifouling properties of PS/AG membranes were tested using different bacterial strains: *Staphylococcus aureus* (*S. aureus*) as a model of Gram-positive bacteria and three other Gram-negative bacteria, namely *E*. *coli*, *Klebsiella pneumonia* (*K. pneumonia*) and *Pseudomonas aeruginosa (P. aeruginosa).*

## 2. Materials and Method 

### 2.1. Materials 

PS with molecular weight of 35 kDa; AG (approximate molecular weight of 250 kDa); N,N-dimethylacetamide (DMA) and paraformaldehyde were purchased from Sigma-Aldrich (St. Louis, MO, USA); Nutrient broth and nutrient agar were obtained from HIMEDIA (Mumbai, India) and phosphate buffered saline (PBS) from ThermoFisher Scientific (Waltham, MA, USA). Commercial microfiltration PS membranes (PM UP150) were obtained from Microdin-Nadir (Wiesbaden, Germany). 

### 2.2. Membrane Synthesis

Membrane casting solutions were prepared by dissolving PS (16 wt. %) in DMA (84 wt. %). Dope solutions were aliquoted and different quantities of AG were added to cast the PS membranes at the following AG loadings: M1 (0 wt. % AG), M2 (0.1 wt. % AG), M3 (1 wt. % AG), M4 (3 wt. % AG), M5 (5 wt. % AG) and M6 (7 wt. % AG). The casting solutions were subject to a period of 1 h sonication using a Q500 sonicator probe (Thomas Scientific, Swedesboro, NJ, USA) while being stirred using a LabForce digital hotplate stirrer (Thomas Scientific, USA) to allow for efficient dispersion of the AG throughout the solution. The solution was degassed for 2 h in order to eliminate air bubbles. The membranes were cast on a glass plate at room temperature according to standard phase inversion techniques using a Labcoat Master casting system (PHILOS, Gyeonggi-do, Korea). The casting speed was set at 3 m/min and a knife gap height of 200 µm. The glass plate was immersed into a coagulation bath containing deionized water (DW) where the solvent (DMA)/non-solvent (DW) exchange takes place causing the polymeric membrane to precipitate off the glass. The cast membranes were washed and stored in DW.

### 2.3. Characterization of Membrane Surface and Cross Section 

Field emission scanning electron microscopy (FESEM) (FEI Versa 3D dual beam, ThermoFisher Scientific, Waltham, MA, USA) was used to examine the surface and cross section of the synthesized membrane samples. Liquid nitrogen was used to splinter the samples and all samples were sputtered using gold. 3 kV vacuum conditions were used throughout in order to yield high-resolution images. 

### 2.4. Membrane Porosity, Pore Size and Flux 

Membrane porosity was evaluated according to the gravimetric method [32]. To that end, membrane samples were cut to a standard size and the initial mass of each sample was recorded while wet. The samples were incubated overnight in an oven set to 50 °C. The next day the dry mass for each sample was recorded. Multiple samples for each membrane were used in order to obtain an average set of total porosity values for the fabricated membranes, calculated according to Equation (1) below:

(1) $$\varepsilon \;\left(\%\right)=\;\frac{w_{w}-w_{d}}{A\times l\times \rho }\;\times 100\%$$

where *W_w_* and *W_d_* represent wet and dry membrane sample masses respectively, *ρ* signifies the density of the DW at 25 °C water (taken as 998 kg/m^3^), *A* is the surface area of the membrane samples (m^2^) and *l* is the membrane sample thickness. 

The membrane average pore size was consequently calculated according to the Guerout-Elford-Ferry equation shown below:

(2) $$r_{m}=\sqrt{\frac{\left(2.9-1.75\varepsilon \right)8\times \eta \times l\times Q}{\varepsilon \times A\times \Delta P}}$$

where ε indicates the total porosity, *Q* represents the volume of DW (permeate) (m^3^/s), *η* is the viscosity of DW at 25 °C (taken as 8.9 × 10^−4^ Pa·s) and Δ*P* is the operating pressure (typically 1 bar). 

In order to evaluate the DW flux for each synthesized membrane, a dead-end stirred cell (Sterlitech HP4750X;Sterlitech Corporation, Washington, USA) pressurized under nitrogen gas, was employed. The permeate flux (*J*) was determined using Equation (3):

(3) $$J=\frac{Q}{A\;\times \;T}$$

where *Q* represents the total volume of permeate collected in time *(T)* and *A* represents the effective cross-sectional area of the membrane exposed to filtration (m^2^). Triplicate tests were conducted for each membrane in order to obtain an average set of results. 

### 2.5. Hydrophilicity and Membrane Surface Charge

A KRÜSS DCA-25 drop shape analyzer (KRÜSS GmbH, Hamburg, Germany) was used to determine the contact angle for selected membrane samples in order to evaluate the hydrophilicity of the synthesized membranes. The volume of each water droplet was set at 2 µL and five readings were taken at randomly chosen sites on each membrane sample. Average values were calculated for each membrane with the standard deviation shown as error bars for each membrane on the plot.

The surface charge for the membranes was determined using a SurPASS™ 3 electrokinetic solid surface analyzer (Anton Paar, Graz, Austria). Triplicate samples for each fabricated membrane were cut to size in order to be placed on the sample cell holder, whilst a 100 µm gap was maintained in between the membrane samples with a streaming potential of 1 mM KCl solution was measured. Zeta potential values were obtained according to the Helmholtz-Smoluchowski equation [33] for a range of pH values at room temperature using 0.1 M NaOH and 0.1 M HCl buffers to alter the pH of aqueous KCl solutions. The average zeta potential values with their respective standard deviations were plotted graphically.

### 2.6. Membrane Biofouling Tests

The antimicrobial properties of the membranes were evaluated using a collection of Gram-positive and Gram-negative bacteria (Table 1). For these experiments, the bacterial suspensions were prepared from an 18 hold nutrient broth culture for each bacterial strain and the bacterial cell density was measured at an optical density (OD) of 600 nm (Novaspec Plus Visible spectrophotometer Amersham Biosciences, GE Healthcare, Chicago, Illinois, USA). The membranes were disinfected by 70% ethanol, dipped in sterilized water, dried in a laminar flow hood and then immersed for 10 min in the bacterial suspension adjusted at 2.4 × 10^7^ colony forming units (CFU)/ml in nutrient broth. Using sterile forceps, the membranes were gently placed on nutrient agar plates and placed in an incubator at 37 °C for 24 h. An additional test was performed using *E. coli* suspension with overnight incubation, keeping the same conditions as the above. 

In order to prepare the bacterial cells for SEM characterization, paraformaldehyde was employed as a cross-linking fixation agent to preserve the cells morphology [34,35]. After incubation of the membranes with bacterial cells, the membranes were removed from the nutrient agar plates and placed gently on empty Petri dishes and then fixed with 4% paraformaldehyde in PBS for 30 min at room temperature [36]. After fixation, the cells were washed twice in PBS and dried in a laminar flow hood. All the samples were sputter-coated with gold and then imaged under FESEM.

## 3. Results and Discussion

### 3.1. FESEM Images of the Membrane Surface and Cross Sections

**Figure 1:**
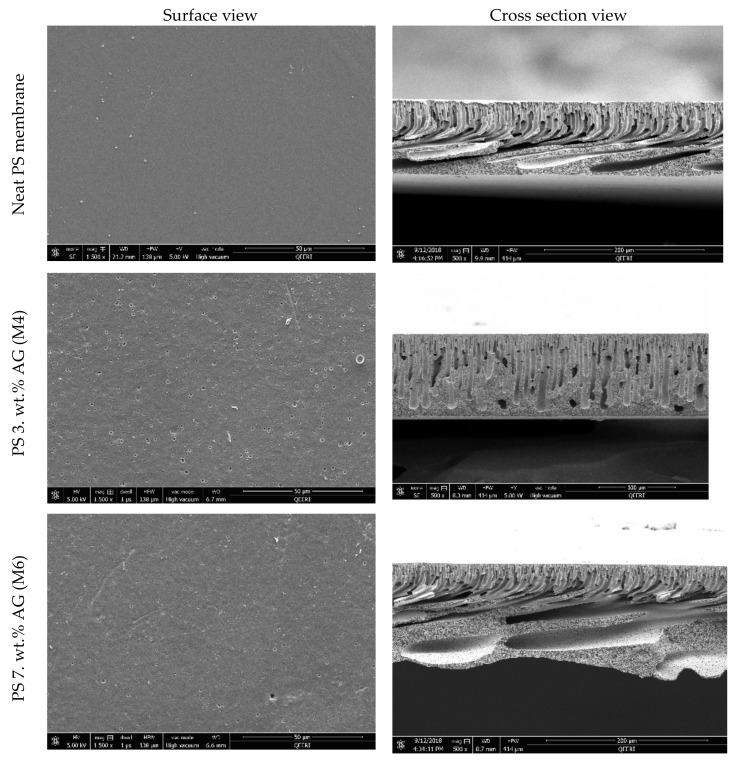
Field emission scanning electron microscopy (FESEM) images of polysulfone (PS) membranes with different arabic gum (AG) loadings. Top panel shows membrane surface morphology, while the bottom panel shows cross sectional images of the selected membranes.

Figure 1 shows the surface and cross sectional FESEM images for selected membrane samples. The surface images show a clear difference in morphology, where an abundance of pores is seen at higher AG (wt. %) loadings compared to neat PS membrane samples. Importantly, no defects were visible at the membrane surface in line with other studies which reported similar findings upon addition of AG [31] or other additives such as PVP [39] to the casting solutions. Several studies have reported that AG addition leads to changes in the porous structures of the cast membranes [30,31]. The cross sectional FESEM images of the membrane samples further validate the above findings. An increase in finger like downward projections from the thin surface layer is noticeable. Larger and more numerous macrovoids are seen in the membrane samples at higher AG loadings. These outcomes can be explained by understanding AG’s role in pore formation. Owing to the fact that AG is an amphiphilic substance due to the presence of both neutral and acidic monosaccharides, these residues confer hydrophilic properties. The presence of hydrophilic AG induces higher thermodynamic instability in the doping solution [39,40]. This ultimately leads to faster demixing between the solvent and non-solvent in the coagulation bath. This instantaneous demixing results in membrane surfaces possessing numerous pores and wider macrovoids in the sponge like matrix below [41]. 

### 3.2. Membrane Porosity, Pore Size and Flux 

**Figure 2:**
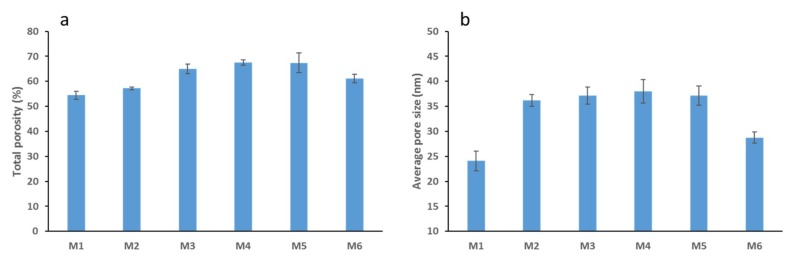
Average values for total porosity (**a**) and average pore size for PS membranes (**b**).

The total porosity data presented in Figure 2a shows an interesting trend where the membrane porosity increases as AG loading increases up to a certain loading threshold. M4 samples which were cast with 3 wt. % AG showed the highest total porosity with a 24% increase from the neat PS membrane samples, closely followed by M5 samples (5 wt. % AG). Interestingly, the total porosity is seen to decrease slightly at the highest AG loading used (7 wt. % AG). These results are in agreement with previous studies which have reported similar findings which have incorporated AG into polymer membranes [30,31] and studies which incorporated inorganic additives [42,43]. The observed trend may be explained in terms of thermodynamics and factors affecting the demixing process. The increase in porosity of PS/AG membranes can be attributed to the hydrophilic nature of AG [44], which acts to accelerate the solvent and non-solvent demixing process which takes place during membrane casting [31]. This acceleration is less evident at higher loadings of AG and other nanofillers investigated [32,43,45] due to the impact the higher loading of the additives has on casting solution viscosity. Viscous polymer solutions experience delayed demixing processes which results in a more dense membrane surface with relatively lower porosity and limited pore connectivity [40,42]. 

The calculated average pore size for all the fabricated membranes increases from 24 nm for the neat PS membrane (M1) to 39 nm for M4 (3 wt. % AG) samples and then decreases to 28 nm for M6 samples which contained the highest loading (7 wt. %) of AG (Figure 2b). The initial increase in average pore size observed can be attributed to AG’s ability to speed up the demixing process described above due to AG’s hydrophilic nature. Several studies have reported that the membrane pore size decreases at maximum loading of nanofiller [30,31,43,45] because of formation of less porous membrane structure at such casting conditions. 

**Figure 3:**
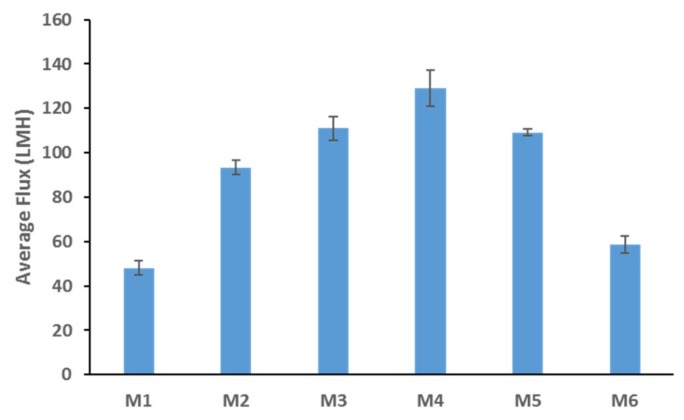
Deionized water DW fluxes for PS membranes cast at incremental loadings of AG.

Figure 3 shows the DW fluxes for the fabricated membranes. The clear trend seen here is similar to the total porosity trend depicted in Figure 2a; where a general increase is followed by a decrease in the flux values. This could be expected since total membrane porosity and pore size are the main parameters affecting membrane flux [46]. M4 samples possessed the highest porosity and pore size values (see Figure 2), while also exhibiting the highest flux readings (see Figure 3). The addition of AG clearly enhances membrane flux (over 100% increase from neat M1 samples to M4 samples). Flux enhancement due to hydrophilic additives has been widely reported in the literature [30,43,45,47,48]. The improvement in flux readings for the PS/AG membranes can be explained by two main reasons. Firstly, the increase in total porosity and interconnectivity of channels present in PS/AG membranes (as evidenced by the FESEM images in Figure 1) lead to better transport of water throughout the membrane. Secondly, the enlargement of sponge-like macrovoid structures seen in the AG-containing membranes observed in the cross-section FESEM images in Figure 1 add to the enhanced transport of water through the basal membrane layer. 

### 3.3. Contact Angle and Surface Charge

**Figure 4:**
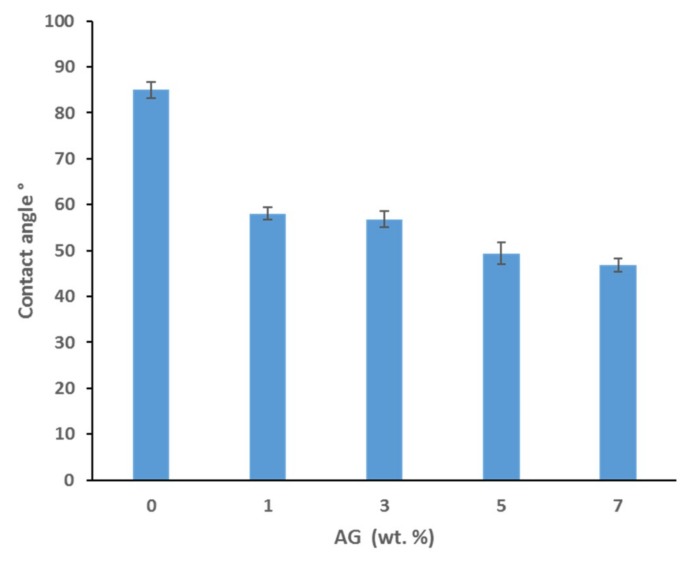
Water contact angles for PS membranes cast with different AG loadings.

Water contact angle measurements were taken in order to evaluate the hydrophilicity of the synthesized membranes. Figure 4 displays the results for the average water contact angles for selected PS membranes with various AG loadings. Smaller water contact angle values indicate higher hydrophilicity and hence wettability of the membranes, whereas larger values indicate more hydrophobic surfaces. A clear trend is seen where the membrane hydrophilicity increases as the AG loading increases, with a 45% decrease in contact angle at the highest AG loading (7 wt.% AG) compared to the bare PS membrane. These findings are consistent with a previous study which yielded results with a similar trend [31]. The hydrophilic nature of AG’s polysaccharide subunits provides a logical explanation for the observable pattern. It could be assumed that the higher loadings of AG in the cast solutions allow for a greater abundance of the hydrophilic side chains which orientate themselves towards the membrane surface during membrane casting, where the boundary with water molecules is found [31]. The addition of other hydrophilic additives, with known pore forming capabilities similarly confer membrane surfaces with increased wettability as indicated by the smaller contact angle values reported [49,50].

**Figure 5:**
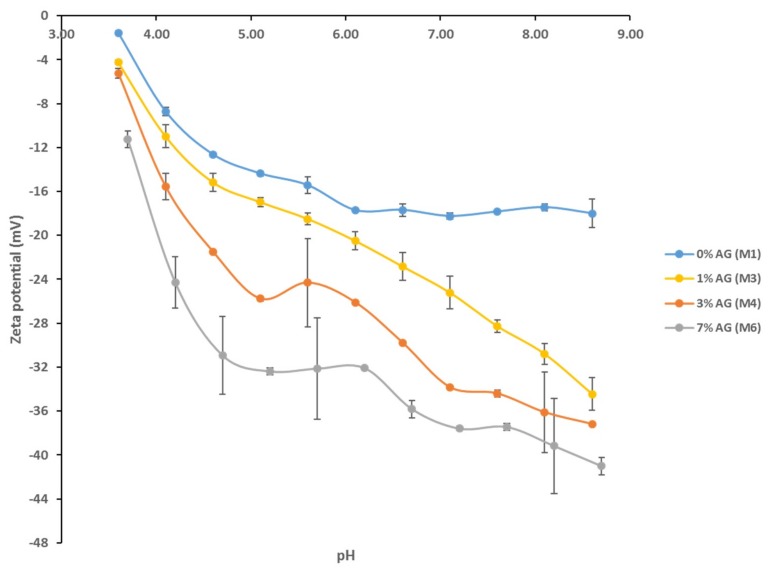
Zeta potential values of PS/AG membranes at different pH.

Figure 5 shows the zeta potential for selected fabricated membranes. There is a common trend exhibited by all membrane samples where the zeta potential values decline as the pH decreases from basic to acidic conditions. Despite some zeta potential readings having large standard deviations (as shown by the error bars), overall there was a notable difference between the different pH scans in terms of the magnitude of negative charge measured at each membrane surface. While the bare PS membrane possesses the slightest negative charge, the M7 sample, which has the highest AG loading, exhibits a far superior negative charge at its surface. For instance, at pH 7, the negative zeta potential increases by 105% for M7 compared to M1 (neat PS) samples. This significant increase in negative surface charge for membranes incorporating AG in the dope solution can be attributed to the hydrophilic residues contained within the AG structure as described above. Specifically, the carboxylic functional groups are responsible for the sharp increase in net negative surface charge as they are known to dissociate across a wide pH range investigated (above pH 2) [31,51]. Indeed, membrane surfaces with increased negative charge are highly desirable when it comes to preventing biofouling processes. Since almost all bacterial species and a wide range of soluble organic foulants (such as proteins at pH values above their isoelectric point) exhibit a net negative surface charge, it has been strongly argued that negatively charged membrane surfaces would decrease bacterial colonization via a repulsion of the similar electrostatic charges [29,52]. 

### 3.4. Antibacterial Properties of PS/AG Membranes Against Gram-positive and Gram-negative Bacteria 

**Figure 6:**
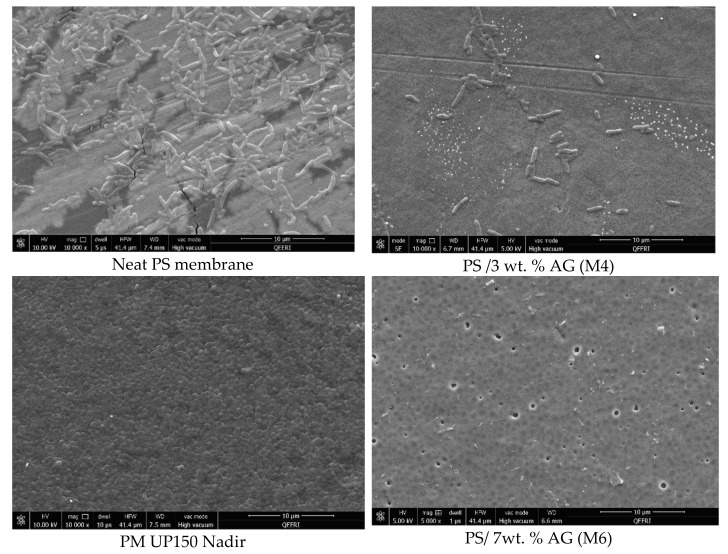
FESEM images of different membrane surfaces after incubation with *E. coli* bacteria.

**Figure 7:**
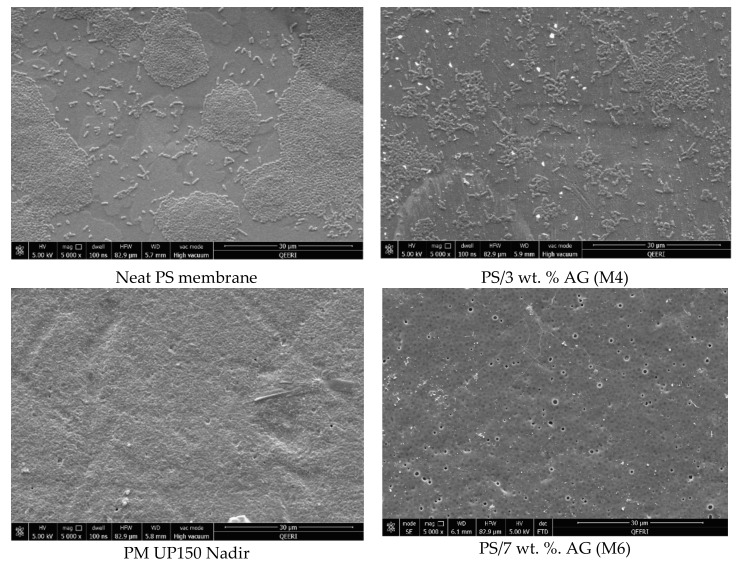
FESEM images of different membrane surfaces after incubation with *P. aeruginosa* bacteria.

**Figure 8:**
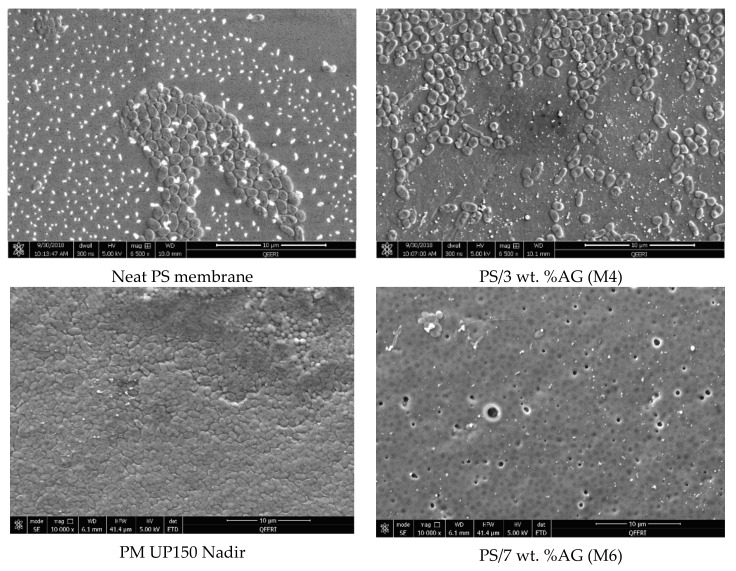
FESEM images of different membrane surfaces after incubation with *K. pneumonia* bacteria.

**Figure 9:**
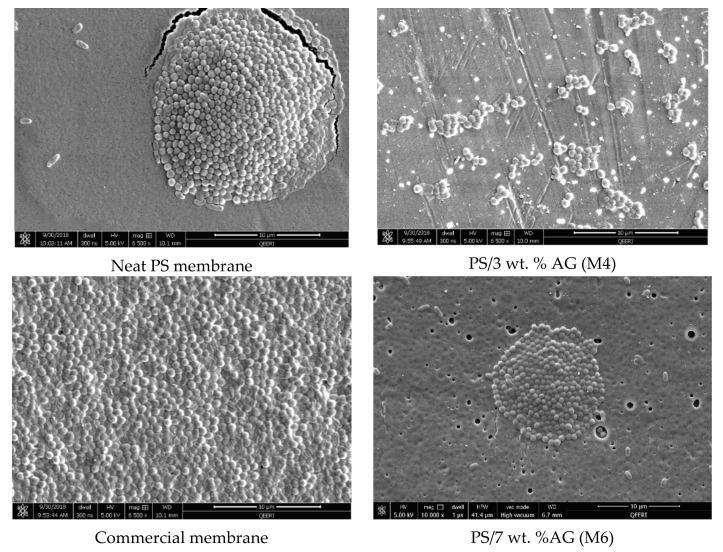
FESEM images of different membrane surfaces after incubation with *S. aureus* bacteria.

**Figure 10:**
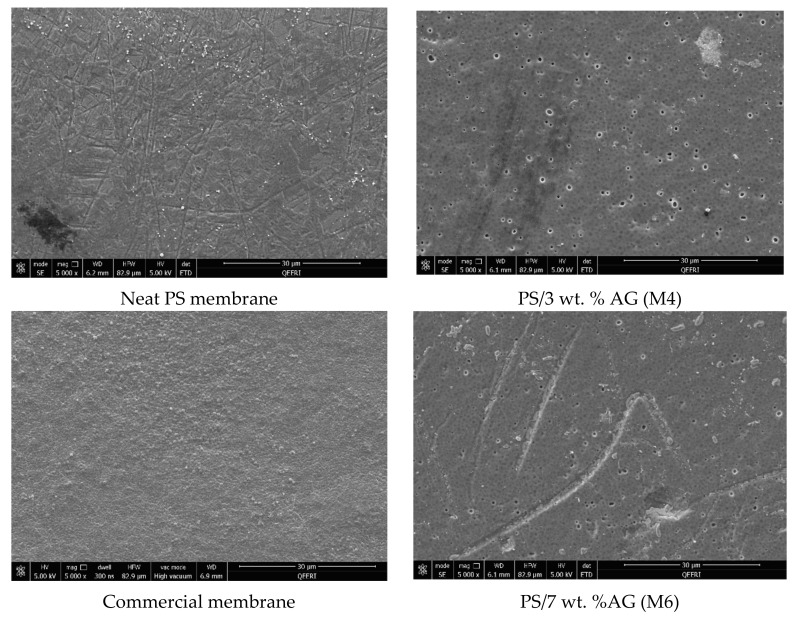
FESEM images of different membrane surfaces after incubation with *E. coli* bacteria overnight.

The antimicrobial properties of PS membranes modified with different loadings of AG were studied by using four bacterial strains (*S. aureus*, *E. coli*, *K. pneumonia* and *P. aeruginosa*) and then compared both with neat PS membranes and PM UP150 (Microdin-Nadir) commercial microfiltration membranes (see Figure 6, Figure 7, Figure 8 and Figure 9). Membranes were also incubated overnight with Gram-negative *E*. *coli* suspension in order to see whether there were any significant differences in bacterial colonization on the membrane surfaces at longer periods of incubation (Figure 10). 

From the SEM analysis, it was observed that the surfaces of the commercial membranes were covered with a layer of bacterial cells for all the strains tested (see Figure 6, Figure 7, Figure 8 and Figure 9). However, all the membranes that incorporated AG showed a significant decrease in bacterial colonization, as seen by the SEM images. Interestingly, the membrane that showed the greatest inhibition was PS membrane with 7 wt. % AG loading (M6), followed closely by M4 sample (3 wt. % AG) (see Figure 6, Figure 7, Figure 8 and Figure 9). 

The neat membrane samples incubated for longer time periods (overnight) with *E*. *coli* suspension showed more widespread bacterial colonization at its surface, compared to its counterpart sample, which was incubated for 10 min (see Figure 6 and Figure 10, respectively). Importantly, both AG-containing membranes incubated for 10 min and overnight showed no essential difference in bacterial adhesion, with samples exhibiting a strong clearance effect at both incubation times. 

The antimicrobial effect exhibited by PS/AG membranes could be related to the incorporation of AG into the membrane matrix. Several studies have investigated the antimicrobial effects of AG, implicating AG as an antibacterial agent [53,54,55,56]. AG is secreted as an exudate by Acacia plants to protect themselves against bacterial and fungal pathogens [57]. The observed decrease in bacterial colonization on PS/AG membrane surfaces could be explained in terms of AG’s antibacterial action or by greater adhesive resistance offered by the membrane surface due to increased hydrophilicity and negative surface charge associated with PS/AG membranes. Indeed, the decrease in bacterial colonization could be due to both mechanisms working together to decrease the number of CFUs observed in the FESEM images. 

Any antibacterial action exhibited by AG would result in damaged or lysed bacterial cells. Duan et al. [43] demonstrated that an ultrafiltration membrane blended with N-halamine grafted halloysite nanotubes displayed potent antibacterial properties against *E*. *coli*. The authors noted significant damage to the morphology of a large fraction of *E*. *coli* cells on the hybrid membrane surfaces [43]. In contrast to their study, the FESEM images shown in Figure 6, Figure 7, Figure 8 and Figure 9 indicate that the surface morphology of the bacterial cells tested in this study were undamaged; namely, spherical cells for *S*. *aureus* and rod-shaped for *E. coli*, *P. aeruginosa*, *K. pneumonia* and *Salmonella*, thus indicating that the PS/AG membrane had no bactericidal effect. 

More likely, it is reasonable to suggest that PS membranes blended with AG exhibit anti-adhesive properties, which prevents the initial attachment of bacterial cells. This is supported by the contact angle and surface charge data shown in Figure 4 and Figure 5, respectively. As already discussed, an increase in hydrophilicity and surface negative charge both act together to inhibit bacterial colonization. It can reasoned that with a highly hydrophilic membrane surface, a film of water molecules will adhere to the membrane surface, thereby disabling bacteria from attaching to the membrane surface and thereby rendering them unable to secrete their EPS. This would ultimately result in a lack of biofilm formation, which is essential for biofouling processes to reach maturity [2]. Similarly, a strongly negatively charged membrane surface would naturally act to repel negatively charged bacterial cells. The presence of negatively charged teichoic acids (for Gram-positive species) and phospholipids and lipopolysaccharides (for Gram-negative species) impart this overall negative surface charge, establishing an electrostatic repulsive effect [58,59]. 

As seen by the FESEM images (see Figure 6, Figure 7, Figure 8 and Figure 9) the control membrane (neat PS membrane) showed some inhibition of bacterial colonization albeit to a much lesser extent than that seen for the PS/AG membrane samples. This unexpected result could be due to the fact that some of the DMA solvent remains in the membrane pore structures after phase inversion membrane synthesis. Since DMA is a known toxic agent to cells [60] it would be reasonable to assume that any antibacterial effects observed in the neat membrane could be due to residual DMA at the membrane surface. 

Interestingly, even at the highest AG loadings, a few bacterial colonies can be observed for the model Gram-positive *S. aureus* bacteria (see Figure 7). This peculiarity could be reasoned in one of two ways. Firstly, it is well known that bacteria are capable of developing resistance towards antimicrobial agents in order to survive by using different mechanisms, which include enzymatic degradation of antimicrobial compounds, alteration of bacterial proteins (antimicrobial target) or the alteration of their bacterial membrane permeability towards antibiotics [61,62,63]. On the other hand, casting membranes using high loading of nanofillers or additives such as AG can lead to agglomeration of the AG and hence resulting in an uneven and non-homogenized distribution within the membrane matrix [64]. As already discussed, the presence of AG acts to increase both hydrophilicity and the magnitude of negative charge of the membrane surface. Surface areas lacking AG would not possess such enhanced features, thereby inactivating the anti-adhesive properties exhibited by PS/AG membranes.

## 4. Conclusions

PS membranes blended with varying loadings of AG were synthesized and characterized using FESEM, contact angle and zeta potential techniques. FESEM images for membrane surfaces indicated the introduction of visible porous structures for membrane samples, which incorporated AG. Total porosity, membrane flux, hydrophilicity and negative surface charge were significantly enhanced for PS/AG membranes compared to bare PS membranes. Such improvements were attributed to the presence of AG, whose amphiphilic nature confers both hydrophilic properties and negatively charged moieties. Together, these characteristics act to decrease bacterial attachment at membrane surfaces, ultimately leading to lower levels of biofouling with *S. aureus, E. coli*, *K. pneumonia* and *P. aeruginosa* bacterial species. All four strains of bacterial species tested showed a similar trend whereby bacterial colonization was almost eliminated for PS/AG membranes synthesized at both 3 wt. % and 7 wt. % AG loading in the casting solutions. In contrast, the commercial and neat PS membranes were unable to inhibit bacterial colony formation. Importantly, a significant decrease in bacterial CFUs in PS/AG membranes was observed for both Gram-positive and Gram-negative bacteria, indicating that the synthesized PS/AG membranes were able to limit bacterial growth for both bacterial models tested. The inhibition of bacterial colonization at PS/AG surfaces was attributed to the enhanced hydrophilicity and membrane surface charge, which contributed in the prevention of bacterial colonization on the fabricated membrane surfaces. These results point to novel membranes capable of significantly minimizing their biofouling rendering them as durable materials for a multitude of membrane technologies related to water treatment. 

## Figures and Tables

**Table 1:** Bacterial strains used in this study.

Bacterial Strain	Gram	Origin	Identification
*Staphylococcus aureus*	Gram-positive	Clinical samples	Isolated on mannitol salted agar and confirmed by biochemical crystal test [37] using Biomic V3 (Giles scientific, USA).
*Escherichia coli*	Gram-negative	Sheep rectal samples	Isolated on selective medium CHROMagar™ (BD–Medysinal FZCO, Dubai, and UAE). Then incubated at 37 °C for 18 h. The single typical *E*. *coli* colonies (green color with a smooth surface) were randomly selected and subsequently streaked onto blood agar plates, and then incubated at 37 °C for 18 h to obtain pure single colonies. For further confirmation, the colonies were transferred onto MacConkey agar plates (BD-Medysinal FZCO) and then blood agar plates (BD Medysinal FZCO), followed by an indole spot test (Remel, Thermo Fisher Scientific, Lenexa, KS) for lactose fermenter isolates and biochemical reactions using Crystal ™ Enteric/ nonfermenter id KIT, BD [38]. Results were interpreted by means of Biomic V3 (Giles scientific, USA).
*Klebsiella pneumonia*	Gram-negative	ATCC reference strain	*K. pneumonia* ATCC, 13883 (Thermoscientific, Kent, UK)
*Pseudomonas aeruginosa*	Gram-negative	ATCC reference strain	*P. aeruginosa* ATCC, BAA-1744 (Thermoscientific, Kent, UK)
